# Our School System

**Published:** 1871-10

**Authors:** 


### HALL'S

### JOURNAL OF HEALTH.

Vol. XVIII. — OCTOBER, 1871. — No. X.

### OUR SCHOOL SYSTEM.

It may safely be said, that nine teachers out of ten do not know the main object of children being sent to school.

It is quite as safe to say, that not one school in a thousand is conducted properly.

In not one school in ten thousand are the proper things taught.

The chief object in sending children to school is not to give them information, intellectually considered, but to discipline the child's mind to think for itself; real information is to be acquired after school days are ended, by this self-thinking, and comparing, and judging. The pert folly, the self-conceit of boys and girls about eighteen, when they begin to think of leaving school, is proverbial. Young people of that age know everything, nothing is doubtful to them; their poor old parents know nothing in their estimation, and are regarded with a kind of pitying contempt. "Father," said one of these Solomons, just from college, "I can prove to you that there are three chickens on the table; you said there were two."

"How do you make it out, my son?"

"Why, it is ever so easy, father. This is one chicken, and that is two; and don't one and two make three?"

"That's a fact, my son. I will take number one, let your mother have number two, and you can have the third one. I hope it will be tender and easily digested, and no doubt you will have a good appetite for breakfast, for I don't intend you shall eat anything else for supper but that third chicken."

A child is educated only in proportion as he learns to think things out himself; and it is from the failure to come up to this point, that we often find to our sorrow that our children know things "by rote," by memory, and are only mechanical scholars.

### METHOD OF STUDY.

Three hours in the forenoon, two hours' interval, and two hours' more study in the afternoon, and not a moment's study of lessons except in the school-room: let all the other time of the waking existence of each day be expended in muscular activities, in moderate work or recreation, involving motion of some kind. No child should be sent to school before seven years of age, and up to eighteen no child ought to be allowed to continue at brain work, involving intellectual labor, longer than the five hours named.

Up to eighteen years, every child should be allowed ten hours to be in bed. They may not require ten hours' sleep, but time should be allowed to rest in bed, after the sleep is over, until they feel as if they had rather get up than not. It is a very great and mischievous mistake for any person, old or young, especially children, and feeble or sedentary persons, to bounce out of bed the moment they wake up; all our instincts shrink from it, and fiercely kick against it. Fifteen or twenty minutes spent in gradually waking up, after the eyes are opened, and in turning over and stretching the limbs, does as much good as sound sleep, because these operations set the blood in motion by degrees, tending to equalize the circulation; for during sleep the blood tends to stagnation, the heart beats feebly and slow, and to shock the system by bouncing up in an instant and sending the blood in overpowering quantity to the heart, causing it to assume a gallop when the instant before it was in a creep, is the greatest absurdity. This instantaneous bouncing out of the bed as soon as the eyes are opened, will be followed by weariness long before noon. Both the brain and body of the young are feeble and watery, and capable of but little endurance. Witness how soon little children get tired of one thing, even of rest. The mother is always complaining that she can't keep her children quiet a minute; put them at anything which they greatly desire to do, and before you know it they will be off at something else: it is because no organ of the brain, no muscle of the body, is strong enough for exertion except for a very short time. Hence both brain and muscle work should be alternated frequently; the overwork of one is as unphilosophical and as criminal as the overwork of the other.

After four or five hours a day have been spent in study, our daughters could, with very great advantage, all through their school course, be required, as their portion of muscular activities, to spend two hours daily in sewing, two in cooking, and two in tidying up the house, such as making beds, sweeping floors and carpets, arranging furniture, and, what is necessary in every household, planning and practicing economies so as to make the most of everything: how to turn and alter various garments; how to make a small amount of food answer the purpose of a good dinner; how to utilize the scraps and pieces of articles left from one meal so as to serve in good part for the next, that "nothing be lost," as was the object of the Master, who came to "seek and to save mankind;" even He did not consider it beneath his great mission to take care of remnants of bread and fish.

It would be well worth a parent's care to spend an hour or more every day, all through childhood, in inculcating lessons of economy in saving things, — in saving all the wastes of the family, if such an expression be allowed. No intelligent mind can doubt that the wastes in the kitchen have bankrupted many a man; that the wastes in clothing, and carelessness in taking care of dresses and other garments on the part of wives and daughters, have kept many a man struggling like a galley slave all his life, to keep his head above water. How many a mother's heart has been pained, after "working her very eyes out" to get up a good dress for her daughter, in the hope of enabling her to make a good appearance, to find after the very first wearing that a rent, a grease spot, a fruit stain, or some other mishap, which a little care could easily have avoided, has rendered it almost entirely useless. At other times a handsome dress, after having been worn the first time, is huddled into a drawer or trunk, to be taken out for the second wearing with as many wrinkles in it as if it had been worn a year. Many girls think that when they get a new dress, it must be worn on every occasion, whether to ride in a carriage or rail car, in dust or sun or rain.; whether to ride at night to a show, or walk a few yards to a reception, all the same. Thus it is some young ladies never appear dressed well, however lavish their expenditures, and on every invitation to a party or a wedding it is the same exclamation, "I've nothing to wear;" that is, nothing fit to wear; which is very true, because nothing is taken care of; while in other cases, girls who have had worthy mothers, with one tenth the expenditure, can bring out dresses, seemingly "bran new," which were made five years before. Surely it would not be time lost if two whole years out of the first eighteen were expended in teaching young girls how to take care of their clothing; but instead of these practical, every-day things being taught in our young ladies' boarding and day schools, time is frittered away in the mere skimming of a dozen different theoretical studies, without the thorough mastering of a single one. A thorough knowledge on the part of our daughters of reading, writing, arithmetic, and grammar, with the domestic instruction above suggested, would add incalculably to the thrift, happiness, and moral elevation of any community.

In the management of the family it is the husband's duty to provide, and the wife's to economize. Many wives have to learn it in the end, who, had they known it in the commencement of married life, would have saved themselves, their husbands, and their children, infinite sorrow.

It is the stock in trade of many writers for the papers to cast slurs on the daughters of the rich about their frivolity and extravagance, but a multitude of cases can be pointed out any day in New York, and in Fifth Avenue, where women born to wealth — by their economies, voluntary and from principle, from a sense of duty — have in times of mercantile disasters nobly turned their energies and their coöperations to the saving of "the house."

On the occasion of a panic, one of our merchant princes came home late one evening, bearing the terrible intelligence to his wife that his failure was inevitable next day; that all his resources were exhausted, and the financial disasters were so general that he could not raise among his friends another dollar, and that he needed a large sum. His wife heard him with extraordinary composure, and quietly asked him how much he needed; and taking down the family Bible, she opened it, and turning over a leaf, found a hundred-dollar bill, and another, and another, until more were counted out than the amazed husband wanted; and on inquiring of her where she obtained so large an amount of money, she said that in prosperous times he had given her such a liberal amount for household and personal expenses, that she was enabled by judicious economies in food and clothing to lay aside a considerable amount every week, and, knowing the fickle character of mercantile life, she thought that it might answer a good purpose to save as she had done.

We know a lady in Fifth Avenue, born to a fortune, her husband a man of wealth, who is constantly supplied with more money than she can use, and regularly returns the surplus. How many daughters and wives among the common classes can be found in a million, who would fail to spend every cent of their "allowance" for dress?

Within a week we met a New York family of four daughters at one of the most costly watering-places in the country. They had been from home several months. They lived at home in their own house, worth eighty thousand dollars, while the generous and jolly father counted his fortune by hundreds of thousands, and yet these daughters, by the teachings of their worthy and lady-like mother, cut and made all their own dresses, and had done so from childhood.

We can point to many women in this city who were raised in affluence, but becoming poor, they have heroically turned their hands to their own support and that of their families, and have nobly succeeded. Let then these two things be borne in mind: that the rich have become rich by judicious economies and self-denials, and by the practice of these things they remain rich; and by teaching these things to their children their good fortune is perpetuated, or in case of reverses which no human foresight could prevent, their education has given them the ability and the self-reliance to hew their own way through the world with triumphant success. But there are very few rich people, not one in a thousand; hence the teachings of this article are for the masses. They are for the remaining nine hundred and ninety-nine families, whose daughters should be educated according to the principles first laid down, educated for the practical duties of domestic life.

